# Motivation and Barriers to Research among Nursing Professionals in Southeast Spain

**DOI:** 10.3390/healthcare10040675

**Published:** 2022-04-02

**Authors:** Carmen Ramón, Bruno José Nievas-Soriano, Jessica García-González, Raquel Alarcón-Rodríguez, Mar Requena-Mullor, David Lozano-Paniagua

**Affiliations:** 1 Faculty of Health Sciences, University of Almería, 04120 Almería, Spain; carmen_rg_2@hotmail.com; 2 Department of Nursing, Physiotherapy, and Medicine, University of Almería, 04120 Almería, Spain; jgg145@ual.es (J.G.-G.); ralarcon@ual.es (R.A.-R.); mrm047@ual.es (M.R.-M.)

**Keywords:** attitude, motivation, barriers, nursing professionals, research

## Abstract

Background: Nursing research promotes quality care and is essential. Thus, it is important to acknowledge the main motivations and barriers that nursing professionals find in their work, the aim of this study was to establish the main aspects that motivate and make nursing research difficult, for nursing professionals; Methods: a descriptive cross-sectional study was carried out on 91 nursing professionals. A validated structured questionnaire composed of 42 items that defined five domains was used. Descriptive and bivariant analyses were performed; Results: the highest scores were obtained for the domain of Knowledge and Preparation (33.79 ± 3.38), while the domain of Available Resources and Support obtained lower mean values (22.60 ± 5.61). Significant differences were found in two domains: Knowledge and Preparation and Professional Development domains, when regarding the service in which the participants were working (*p* < 0.05); in the Available Resources and Support domain, when regarding sex (*p* < 0.05), in the Motivations domain, regarding the number of children (*p* < 0.05); Conclusions: nursing professionals show a positive attitude towards nursing research. The nurses find motivation in their work environment, in the economic incentives, or in the possibility to improve their curriculum vitae. The main barriers are the lack of time, the lack of institutional support, and the lack of training, especially in languages, such as English. These findings could be useful to design programs to overcome these barriers.

## 1. Introduction

Nursing research has been defined as a scientific process that validates and improves existing knowledge and generates new knowledge that influences the nursing practice, directly or indirectly [1]. Research is the basis of any profession. Research is important to develop evidence-based nursing, based on quality. This is essential for the patients but also the caregivers, the healthcare professionals, and the health system [2,3]. In the last decades, the evolution of nursing research has been remarkable, although the conditions have not always been optimal. Some of the barriers found to perform research in nursing include clinical setting characteristics, lack of access to resources, lack of time, lack of colleague cooperation, no authority to modify care protocols, little managerial support, lack of research skills, poor team working, and no incentives [4,5]. In Spain, the historical circumstances in the second half of the twentieth century limited the access to research in nursing, but the development of the university nursing degree allowed a considerable increase in the number of scientific publications [2] due to a change of focus of the professional activities of the nurses, the appearance of new scientific nursing journals and more university-associated professionals, and larger budgets for researching [6].

In this sense, the Spanish Law 44/2003, on the Organization of Health Professions, recognized the importance of the research work for the health professions in this country. However, it was not until 2008 that research work in nursing was recognized as one of the main competencies of the profession, with the purpose of basing the actual clinical practice on the existing scientific evidence [7]. Consequently, in these last years, the number of peer-reviewed articles regarding nursing has increased significantly, coinciding with an increase in the number of nursing doctorates in Spanish universities, who have been considered the main axis for the promotion of this research [2,7].

Nursing research is essential for the development of the nursing profession. For some authors, this is because knowledge based on tradition and experience is being replaced by evidence-based knowledge [1,8]. Clinical practice guidelines, algorithms, and care plans help to transfer evidence-based knowledge to the clinical setting, being useful not only as educational materials for professionals but also allowing to give clinical feedback to researchers [1,9]. In developing countries with high morbidity, research investments could indeed be critical to enrich the healthcare policies and to offer quality nursing care. Nursing research results in evidence-based service delivery and policy recommendations [10]. Investments enrich the effectiveness of global health policy, improving the quality of care [11].

In the Quality Plan for the National Health System of the Spanish Ministry of Health [12], the promotion of research among health professionals is proposed as one of the main objectives of this plan. In this sense, the International Council of Nurses views research as a reflective, critical, and realistic form of practice that enables nurses to view problems from a different perspective, as the research-based practice is essential for good professional development [13]. Nevertheless, the development of research-based nursing is still scarce, mainly due to the fact that research is still performed for the sake of obtaining an academic degree [14]. Albeit some authors have described different theoretical frameworks and instruments for evaluating critical thinking in nursing and education [15], we have not found a model developed explicitly for Spanish nurses.

In Spain, research is an integral part of the nursing degree curriculum and the nurses’ clinical practice. The requirement for research for health professionals and nurses in Spain is similar to the requirement for Evidence Based Practice for health professionals and nurses in the United States. During their professional career, research is required to obtain professional positive evaluations, which are required to level up their career and improve their salary. Therefore, all of them must perform research during their professional career, and research is an essential part of their daily work, Spain being among the most productive countries [16]. Nevertheless, not all nurses are equally qualified to perform research.

Although the research in nursing offers plenty of opportunities, such as establishing research networks with nursing care research groups, centralizing and disseminating scientific nursing information and knowledge, broadening participation, and taking advantage of synergies [8], there are still some barriers, such as the lack of time, resources, motivation, funding, or even knowledge gaps. To overcome these difficulties, it is essential to find support and finance [8,17]. Thus, this research tries to better acknowledge some of these aspects, such as the lack of knowledge about the research in nursing, the resources, the motivations, and the interests of the nursing professionals, regarding the nursing research. As research is the base of professional development, our findings could be useful for the future of the research in this profession: the description of the motivations and the interests of the nursing professionals, and the main barriers regarding the nursing research may help nursing professionals, researchers, and healthcare stakeholders to make decisions that contribute to developing nursing-related scientific production [18,19].

Therefore, the aim of this research was to acknowledge nursing professionals’ main motivations to perform research and to identify the main barriers that professionals find when trying to undertake research, and to show its importance in professional development.

## 2. Materials and Methods

### 2.1. Study Design and Participants

A multicentric cross-sectional descriptive study was performed. According to other authors [20], the Epi Info™ App (Epi Info™, Division of Health Informatics and Surveillance, Center for Surveillance, Epidemiology and Laboratory Services, GA, USA), available at https://www.cdc.gov/epiinfo/esp/es_index.html (accessed on 14 February 2022), was used with the following parameters: population size 3071 (nurses working in Almeria); expected frequency of 93.6%; confidence level of 95%; margin of error within 5%. These parameters indicated a required sample size of 89 participants.

A total of 91 nursing professionals from healthcare centers and the hospitals of the province of Almeria, Spain, accepted to take part in the research. The inclusion criteria were the following: nursing professionals from a healthcare center or a hospital of the province of Almeria, that were active and working, and that could read and speak Spanish. The nursing professionals who were not working, or who did not speak Spanish, were excluded.

### 2.2. Variables and Measurement

A validated questionnaire named “Questionnaire on motivation towards research in nursing” was used to evaluate the motivation for performing research among nursing professionals [21,22]. This tool contained 42 items that defined five domains: personal and professional time management; knowledge and preparation; available resources and support; professional development; and motivations (Table S1). No translation or transcultural adaptation was required, as the questionnaire was previously validated in Spanish for nursing professionals [22,23]. Responses for the items were five-point Likert scales, being 1—totally disagree, and 5—totally agree. The scoring range was from 8 to 40 for domains 1, 2, 3, and 5; for domain 4 was from 10 to 50. The global score was the sum of the five domains and its range was from 42 to 210. Higher scores meant that the answers of the participants were more in concordance with the abilities defined in the different domains. No cut-offs were defined, as they were not required to analyze the answer. The reliability of the questionnaire was evaluated using Cronbach’s alpha test, with a value of 0.68. The following demographical features of the participants were also collected: sex, age, number of children, level of education, employment situation, work center, working service, working years fulfilled.

### 2.3. Data Collection

A website version of the questionnaire was developed using the Google Forms platform (Google LLC, Mountain View, CA, USA) and shared with the population study through the app WhatsApp (Facebook, Menlo Park, CA, USA). Potential participants received an invitation to take part in the research, followed by a link. Once they accessed the link, the following information was shown: a description of the study and its main aims, instructions for fulfilling the questionnaire, the estimated duration of the survey (less than 5 min), and information about the use of the collected data. Participation was voluntary. Informed consent was shown on the initial page of the questionnaire. It was compulsory to accept it, to take part in the research. Only fully completed questionnaires were admitted. Personal data were not collected. Google Forms generated a spreadsheet with the answers to the questionnaires, that were kept on their server. The data collected were used, without any manipulation or statistical correction, to perform the statistical analyses. All data were collected between February and March 2021. No questionnaires needed to be discarded.

This research was approved by the Research and Ethics Committee of Nursing, Physiotherapy, and Medicine Department of the University of Almeria (Almeria, Spain), with approval number EFM 123/2021. The participants were informed about the main aims of the research, and informed consent was shown at the beginning of the questionnaire. The questionnaire did not collect personal information, and all the data collected were managed according to the Spanish Law on the Protection of Personal Data (15/1999) and the Organic Law of Personal Data Protection and Warranty of Digital Rights (3/2018).

### 2.4. Statistical Analyses

Statistical analyses were performed using SPSS version 26 (IBM Inc., Armonk, NY, USA). A descriptive analysis was performed for demographical variables. Percentages and frequency distributions were calculated for categorical variables. For quantitative variables, mean and deviation were calculated. The Kolmogorov–Smirnov test was used to analyze the distribution of the quantitative variables. The Student’s *t*-test and the ANOVA test were used to compare the means in normal distribution variables, and the Mann–Whitney test and the Kruskal–Wallis test in independent variables. The Spearman correlation test was used to establish the correlation among the variables. A significance level of 95 % was considered.

## 3. Results

### 3.1. Demographical Features

A total of 91 nursing professionals took part in the research. They were aged between 22 and 61 years, with a mean age of 33.79 years. Most of them (84.6%) were women, and 71.4% of the participants had no children. Of the participants, 91.2% were working exclusively, and 8.8% were working and studying. A total of 52.7% of the participants had a degree education level, 45.1% had postgraduate education, and only 2.2% had a doctorate. A total of 23.1% of the participants worked at the intensive care unit, 20.0% worked in the hospital conventional ward, and 18.7% worked in primary healthcare centers. When considering the services where the participants worked, 13.2% of them worked in the labor room, 6.6% worked in the emergency service, and 5.5% in the COVID-19 ward. The rest of the demographical features of the participants are shown in Table 1.

### 3.2. Motivation for Performing Research

The global score of the test and the mean scores of the five domains of the questionnaire are shown in Table 2. The Knowledge and Preparation domain obtained the highest score, while the lowest score was for the Available Resources and Support domain. The global mean score of the questionnaire was 146.5 points.

The results of the bivariate analyses of the different domains and the global score of the questionnaire, regarding the demographical variables, are shown in Table 3. No statistical significative differences were found in the global score when analyzing the demographical variables. When analyzing the domains, significant statistical differences were found in some domains: women scored higher on the domain Available Resources and Support. The highest scores in the Motivation domain were from participants with higher education levels. Two domains, Knowledge and Preparation, and Professional Development obtained different scores depending on the service where the participants were working at the moment of taking part in the research. In the Motivations domain, the participants with no children scored higher. A negative correlation was found between the number of children and this same domain, and the Knowledge and Preparation one. No differences were found, for the rest of the demographical variables analyzed.

### 3.3. Main Barriers for Performing Research

According to the answers of the participants (Table S2), the main barriers found were the following: lack of formation about research (98.9%), not knowing other languages, such as English (96.7%), lack of time (80.2%), high loads of work (79.2%), and lack of institutional support (71.3%).

## 4. Discussion

The study aimed to establish the main aspects that motivate and make research difficult for nursing professionals and to show its importance in professional development. To perform that task, a validated questionnaire was used. The global score of the participants of our research was like those obtained by other authors [23], who differentiated between nursing professionals who worked exclusively in researching, from those who worked in hospitals or in primary healthcare.

### 4.1. Analysis of Demographical Variables

Regarding the demographical variables, no significant differences were found when analyzing the global score of the questionnaire. Significative differences were found in the Available Resources and Support domain when regarding sex, as women scored this domain higher than men. This is in concordance with the finding of other authors, who concluded that there were significant differences in the development of a research profile, according to the sex, as more researchers are men [23,24]. This inequality could be mirroring the general society, where the women have more familiar commitments and less time for researching [23]. In our research, no differences were found according to the age of the participants, as in the findings of other authors [25]. Nevertheless, other articles describe a positive correlation between the age of the participants and their research skills [26].

In the Motivations domain, significant differences were found regarding the education level, as the participants with a doctorate showed a higher level of motivation to perform research. This finding is in concordance with other publications [23,24]. For some authors, a higher education level influences professional development and the commitment to perform research [27].

We found no differences regarding the years of work, while other authors concluded that there is a positive correlation between this factor and the skills of the participants, to perform research [27]. Significative differences were found, in the Knowledge and Preparation domain, when regarding the different services where the participants worked. The highest scores were from the participants who worked in the delivery room, the COVID-19 ward, and general hospital wards. The lowest scores came from the nursing professionals of emergencies. We have not found other studies that have evaluated this specific aspect, although some authors state that the nursing professionals that work in high-complexity hospitals and those with stable contracts have more interest in performing research [28]. Other authors found differences between the nursing professionals that worked in ICU and Emergencies when evaluating the barriers to performing research, as the professionals from Emergencies found more barriers [25,28].

Finally, we found that the nursing professionals with no children scored higher in the Motivation domain when compared to professionals with children. We found a negative correlation between the two domains, Knowledge and Preparation, and Motivation, regarding the number of children of the participants. As described by other authors [28,29,30,31], this finding seems logical as nursing professionals with children have less time to devote to research. Therefore, their knowledge and preparation in this field could be lower and their motivation to perform research can be affected.

### 4.2. Motivations for Performing Research

In our study, 80.2% of the participants stated that performing research improves clinical practice. This finding is in concordance with the results of other similar research [16], where the authors conclude that a positive attitude towards research is essential for professional development. We also found interest, among the participants, to perform research. Nevertheless, other authors state that the lack of motivation is a barrier frequently described by nursing professionals [28]. These same authors concluded that the motivation could rise within the next years as many of their participants showed interest in developing future projects.

We found some interesting motivations to perform research. A total of 78% of the participants stated that being motivated in their work helped them to perform research. A total of 72.5% of the participants also mentioned the importance of the economic incentives, and 58.3% pointed out that improving their curriculum vitae was their main motivation to perform research. These findings differ from those of other authors, who concluded that the main motivation was to keep up to date in areas such as the safety of the patients and the nursing cares quality [25]. Other authors conclude that intrinsic motivations are more relevant than extrinsic ones, being the most important one the professional development [27].

### 4.3. Barriers for Performing Research

The analysis of the different domains of the questionnaire showed that the Knowledge and Preparation domain obtained the highest scores, while the Available Resources and Support domain obtained the lowest scores. These results differ from other authors, who state that the main barrier for research is intrinsic factors of the nursing professionals [23]. When analyzing the items of the questionnaire (Table S2), we found that the most mentioned barriers were the lack of time (80.2%) and the high loads of work (79.2%). These two aspects, along with the lack of resources, are proposed by other authors as the main barriers [23,24,25,26,32,33,34]. Other authors also mention the lack of funding [24,33]. The lack of support from the medical staff has been also described as a potential barrier by many authors [23,25,26,28,32,34]. Policies that enable nurses to use more time for research could be also useful [5]. A specific nursing research department could help to improve the aptitude for research among nursing professionals [10,32,35]. Nevertheless, in our research, we did not find that the lack of support from the medical staff was perceived as a barrier.

Almost all the participants of our research (98.9%) answered that nursing professionals need more information about research. This finding has been described by other authors [31] who state that attending compulsory courses about the methodology of research was perceived as appropriate by the nursing professionals. Other authors describe that most of the participants in their research were interested in receiving training to perform research but only a small percentage had received it [24]. Other authors found that almost all the nursing professionals highlighted the importance of research but only a fifth part of them was willing to obtain a doctorate [25].

Another important finding of our research was that 81.3% of our participants answered that nursing professionals are as prepared as other healthcare professionals to perform research. This finding differs from other publications [36], where the nursing professionals declared a lower knowledge level when compared to the medical staff. In other research the professionals defined their ability for research as weak, scoring low their abilities to evaluate the reliability of the research and the access to scientific literature [32]. They only scored high in their ability to evaluate the ethical aspects.

Another finding that must be highlighted is that 96.7% of our participants declared that they did not know other languages, such as English, which could facilitate performing research. This problem has been described by other authors [33,34,37]. In our research, the participants stated that nursing professionals have authority, contrary to other research, where this lack of authority is described as a potential barrier [23,26,31,33,36].

According to our findings, some realistic measures could be helpful to encourage nurses’ research, such as establishing a percentage of their work hours to perform research; setting a minimum number of required publications in journals, among the objectives of the nursing management units of healthcare centers; or offering economic incentives regarding the number of publications in high impact factor journals.

### 4.4. Strengths and Limitations

The main strength of this study is that the participants are anonymous nurses that work in different healthcare centers, so our results reflect their real perceptions about this topic but without the bias of performing personal interviews, which can alter their answers. Another advantage is that the questionnaire was previously validated, which adds validity to our findings. Finally, our research can be a pilot test of future research about this specific aspect of the professional development of nurses.

However, this research also has some limitations that should be considered when interpreting the external validity of the findings. We used the Epi Info™ App (Epi Info™, Division of Health Informatics and Surveillance, Center for Surveillance, Epidemiology and Laboratory Services, Atlanta, GA, USA) to estimate the sample size required and, in web-based questionnaires, some authors have stated that it is usual to have survey view rates of less than 0.1%, and even inferior participation rates [38]. Albeit the participation in our research was higher than this figure, and the sample size was above the estimation calculated, the size of our sample should be considered, as it could limit the generalization of our findings. Another limitation is that the sample was a convenience sample. In web-based surveys, selection bias occurs inevitably [38,39] as users are more likely to respond to questionnaires if they are interested in the topic [40]. We must also consider the differences of the different breeds of nursing professionals that have participated in this research. Some of the services counted on very few participants, so the external validity of the subsequent hypothesis contrast should be considered with caution. Finally, the evaluation of this research has been performed using a quantitative method based on a validated questionnaire, so we have not obtained qualitative information from the participants that perhaps could have been useful. 

## 5. Conclusions and Future Research

Evidence-based nursing is considered one of the main pillars for the development of nursing professionals. Decision making based on scientific knowledge from research and the clinical experience of the health care nursing staff gives nursing staff knowledge and training for the development of their work. Our findings show that, albeit nurses have a positive attitude towards performing research and they consider it an essential part of their work, the percentage of nursing professionals that perform research is still too low.

The nurses declare to find motivation in their work environment, in economic incentives, or in the possibility to improve their curriculum vitae. By contrast, the main barriers are the lack of time, lack of institutional support, and the need for further training, especially in languages, such as English. These aspects hinder their research potential since it is difficult to combine them with their work and complementary studies.

Therefore, healthcare managers should provide adequate resources to facilitate the development of research among nurses, such as time, technical tools, or even English learning. These findings can be useful to design programs to overcome these barriers. Thus, more studies about the motivations and barriers of nurses to perform research are necessary.

For future research it would be useful to use a bigger sample, to compare the differences among the different breeds of nursing professionals, especially if the sample is bigger, and to perform a qualitative evaluation of the motivations and perceived barriers of the professionals. Thus, for future studies, one of the advantages of using a web questionnaire is that bigger sample sizes can be recruited and compared to our research.

## Figures and Tables

**Table 1:** Demographical variables of the participants.

Variable		n	%
sex	men	14	15.4
	women	77	86.4
age		33.8 *	11.2 **
employment situation	working and studying	8	8.8
	exclusively working	83	91.2
education level	graduate	48	52.7
	postgraduate	41	45.1
	doctorate	2	2,2
working years		10.1 *	10.9 **
present working service	intensive care unit	21	23.1
	primary healthcare	17	18.7
	emergencies	6	6.6
	delivery room	12	13.2
	COVID-19 ward	5	5.5
	hospitalization ward	19	20.9
	other	7	7.7
children	yes	26	28.6
	no	65	71.4

* Mean; ** Standard Deviation.

**Table 2:** Score of the domains and global score of the questionnaire.

Domain	Mean	SD	Range
personal and professional time management	30.91	4.87	8–40
knowledge and preparation	33.79	3.38	8–40
available resources and support	22.60	5.61	8–40
professional development	30.63	3.50	10–50
motivations	28.53	5.39	8–40
global score	146.5	11.5	42–210

SD = Standard Deviation.

**Table 3:** Bivariant analysis of the domains, regarding demographical features of the participants.

Variable	Personal and Professional TimeManagement	Knowledge and Preparation	Available Resources and Support	Professional Development	Motivations
sex					
men	32.6 ± 5.1	33.5 ± 4.8	19.1 ± 5.4	15.9 ± 6.5	22.2 ± 8.7
women	30.6 ± 4.8	33.8 ± 3.9	23.3 ± 5.4	17.3 ± 3.6	23.9 ± 5.1
*p*-value	0.13 *	0.56 *	0.01 ***	0.98 *	0.73 *
age	0.14	−0.01	0.09	−0.13	−0.15
*p*-value	0.19	0.99	0.39	0.23	0.15
employment situation					
exclusively working	30.6 ± 4.1	35.7 ± 4.5	19.9 ± 4.6	19.2 ± 3.2	25.1 ± 5.9
working and studying	30.9 ± 4.9	33.6 ± 3.2	22.9 ± 5.6	16.8 ± 4.2	23.5 ± 5.8
*p*-value	0.75 *	0.12 *	0.15 ***	0.10 *	0.44 *
working years	0.10	0.00	0.07	−0.12	−0.11
*p*-value	0.32	0.99	0.49	0.26	0.29
education level					
graduate	31.1 ± 5,2	33.3 ± 3.3	23.0 ± 5.8	16.6 ± 3.9	22.3 ± 5.4
postgraduate	30.4 ± 4,4	34.1 ± 3.4	22.2 ± 5.6	17.5 ± 4.6	24.9 ± 5.9
doctorate	36.0 ± 0.0	38.0 ± 0.0	21.0 ± 0.0	19.0 ± 0.0	30.0 ± 0.0
*p*-value	0.12 **	0.06 **	0.75 ****	0.26 **	0.01 **
present service					
intensive care unit	30.1 ± 4.5	33.6 ± 3.1	21.2 ± 5.2	17.6 ± 3.8	25.0 ± 4.1
primary healthcare	29.6 ± 5.8	32.6 ± 2.1	26.2 ± 6.1	16.5 ± 3.8	22.7 ± 4.8
emergencies	30.2 ± 3.2	30.2 ± 5.9	25.8 ± 5.3	12.3 ± 4.0	17.8 ± 10.5
delivery room	33.2 ± 2.6	36.0 ± 2.0	21.8 ± 4.7	16.7 ± 3.7	26.1 ± 3.2
COVID-19 ward	30.4 ± 4.1	35.4 ± 5.3	20.8 ± 1.1	18.4 ± 3.3	23.0 ± 7.8
hospitalization ward	31.8 ± 5.3	34.5 ± 3.0	20.9 ± 6.0	16.7 ± 5.1	23.9 ± 6.0
other	28.6 ± 6.0	33.1 ± 2.6	23.1 ± 5.6	20.7 ± 1.6	26.3 ± 5.1
*p*-value	0.36 **	0.03 **	0.05 ****	0.02 **	0.34 **
children					
yes	30.9 ± 6.1	32.9 ± 3.0	21.8 ± 5.3	16.8 ± 4.4	21.7 ± 4.9
no	30.9 ± 4.4	34.1 ± 3.5	22.9 ± 5.7	17.1 ± 4.12	24.4 ± 5.9
*p*-value	0.72*	0.06 ***	0.37 *	0.70 *	0.02 *
number of children	0.03	−0.21	−0.06	−0.01	−0.27
*p*-value	0.75	0.04	0.59	0.91	0.01

* Mann–Whitney test; ** Kruskal–Wallis test; *** t-student test; **** ANOVA. For quantitative variables, Spearman correlation was used.

## Data Availability

The data presented in this study are available on request from the corresponding author.

## Supplementary Materials

The following supporting information can be downloaded at: https://www.mdpi.com/article/10.3390/healthcare10040675/s1, Table S1: Domains and questions included in the validated questionnaire (English translated); Table S2: Answer to ítems of validated questionnaire.
