# Supplementary material for: Motivation and Barriers to Research among Nursing Professionals in Southeast Spain
Source: Healthcare (Basel). 2022 Apr 2;10(4):675. doi: 10.3390/healthcare10040675 (PMC9029644; doi:10.3390/healthcare10040675)
Supplement: Supplementary file 1 [file healthcare-10-00675-s001.zip › TableS1.pdf]

Table S1: Domains and questions included in the validated questionnaire (English translated)

Item	**Domain 1: Organisation of personal and professional life: time management**	TD	ED	NA	DA	TA
1	Research requires a lot of time and effort outside working hours.	**1**	**2**	**3**	**4**	**5**
2	My personal life will be negatively affected if I do research work.	**1**	**2**	**3**	**4**	**5**
3	Personal obligations outside working hours are an obstacle to carrying out research work.	**1**	**2**	**3**	**4**	**5**
4	I do not have time during working hours to devote to a research project.	**1**	**2**	**3**	**4**	**5**
5	Carrying out research work during working hours would hinder the performance of my duties.	**1**	**2**	**3**	**4**	**5**
6	The distribution of the working day into shifts makes it difficult to devote time to research.	**1**	**2**	**3**	**4**	**5**
7	In my job, the burden of care is one reason why it is difficult to devote time to research.	**1**	**2**	**3**	**4**	**5**
8	Part of the working time should be spent on research projects.	**1**	**2**	**3**	**4**	**5**
**Item**	**Domain 2: Knowledge and preparedness**	**TD**	**ED**	**NA**	**DA**	**TA**
9	I have sufficient knowledge to carry out a research project.	**1**	**2**	**3**	**4**	**5**
10	The nursing professional should read scientific journals regularly, at least once a month.	**1**	**2**	**3**	**4**	**5**
11	There is not enough training for nursing research.	**1**	**2**	**3**	**4**	**5**
12	The nursing professional must take postgraduate training courses on research.	**1**	**2**	**3**	**4**	**5**

13	Nursing curricula should include research training.	**1**	**2**	**3**	**4**	**5**
14	Knowledge of new technologies (internet, databases, etc.) facilitates research.	**1**	**2**	**3**	**4**	**5**
15	Knowledge of other languages facilitates research.	**1**	**2**	**3**	**4**	**5**
16	Nursing is equally qualified as other health professionals to carry out research studies.	**1**	**2**	**3**	**4**	**5**
**Item**	**Domain 3: Available resources and support**	**TD**	**ED**	**NA**	**DA**	**TA**
17	I receive support and motivation from the nursing management to do research.	**1**	**2**	**3**	**4**	**5**
18	Nursing research receives the same external support (grants, prizes, subsidies, publications, etc.) as that received by other health professionals.	**1**	**2**	**3**	**4**	**5**
19	At my workplace, I have access to sources of information for research.	**1**	**2**	**3**	**4**	**5**
20	Research requires financial resources that are difficult to obtain.	**1**	**2**	**3**	**4**	**5**
21	At my workplace, the rest of the multidisciplinary team would collaborate in a research project in nursing.	**1**	**2**	**3**	**4**	**5**
22	At my workplace, I have access to material resources (paper, computer, software, etc.) to carry out research.	**1**	**2**	**3**	**4**	**5**
23	At my workplace, I have access to scientific nursing literature.	**1**	**2**	**3**	**4**	**5**
24	Nurses have the same economic support as other professionals for research.	**1**	**2**	**3**	**4**	**5**
**Item**	**Domain 4: Professional development**	**TD**	**ED**	**NA**	**DA**	**TA**

25	Research is a function of my professional activity.	**1**	**2**	**3**	**4**	**5**
26	As a nurse, I have the authority to make decisions about patient care.	**1**	**2**	**3**	**4**	**5**
27	Nursing functions are primarily practical and do not need to include research.	**1**	**2**	**3**	**4**	**5**
28	I would be willing to collaborate with other professionals in their research work.	**1**	**2**	**3**	**4**	**5**
29	Research brings benefits to professional nursing practice.	**1**	**2**	**3**	**4**	**5**
30	I do not need research to improve my work.	**1**	**2**	**3**	**4**	**5**
31	Research is a function delegated to me by other professionals.	**1**	**2**	**3**	**4**	**5**
32	In nursing, it is difficult to find interesting fields in which to do research.	**1**	**2**	**3**	**4**	**5**
33	Research improves the recognition of the nursing profession.	**1**	**2**	**3**	**4**	**5**
34	Nursing is not a research-oriented profession.	**1**	**2**	**3**	**4**	**5**
**Item**	**Domain 5: Motivations**	**TD**	**ED**	**NA**	**DA**	**TA**
35	I would like to participate in a research project.	**1**	**2**	**3**	**4**	**5**
36	Being motivated in my work encourages me to do research.	**1**	**2**	**3**	**4**	**5**
37	I feel capable of leading a research project.	**1**	**2**	**3**	**4**	**5**
38	Achieving a higher degree in my professional career is an incentive to research.	**1**	**2**	**3**	**4**	**5**
39	A financial reward would increase research interest.	**1**	**2**	**3**	**4**	**5**
40	Publishing a research paper is not professionally recognized.	**1**	**2**	**3**	**4**	**5**

41	It is not worth researching to increase the score on my CV.	**1**	**2**	**3**	**4**	**5**
42	I am interested in the research work carried out at my workplace.	**1**	**2**	**3**	**4**	**5**

TD: strongly disagree; ED: disagree; NA: disagree not at all; DA: agree; TA: strongly agree.
